# RNA-binding protein p54^nrb^/NONO potentiates nuclear EGFR-mediated tumorigenesis of triple-negative breast cancer

**DOI:** 10.1038/s41419-021-04488-9

**Published:** 2022-01-10

**Authors:** Mengqin Shen, Ruixue Zhang, Wenzhi Jia, Zongping Zhu, Li Zhao, Gang Huang, Jianjun Liu

**Affiliations:** 1 Department of Nuclear Medicine, Institute of Clinical Nuclear Medicine, Renji Hospital, School of Medicine, Shanghai Jiao Tong University, 200127 Shanghai, China; 2 Department of Nuclear Medicine, Qingdao Municipal Hospital (group), 266011 Qingdao, China; 3 Shanghai Key Laboratory of Molecular Imaging, Shanghai University of Medicine and Health Sciences, 201318 Shanghai, China; 4 State Key Laboratory of Oncogenes and Related Genes, Shanghai Cancer Institute, 200127 Shanghai, China

**Keywords:** Bone cancer, Cell growth, Molecular biology

## Abstract

Nuclear-localized epidermal growth factor receptor (EGFR) highly correlates with the malignant progression and may be a promising therapeutic target for breast cancer. However, molecular mechanisms of nuclear EGFR in triple-negative breast cancer (TNBC) have not been fully elucidated. Here, we performed gene-annotation enrichment analysis for the interactors of nuclear EGFR and found that RNA-binding proteins (RBPs) were closely associated with nuclear EGFR. We further demonstrated p54^nrb^/NONO, one of the RBPs, significantly interacted with nuclear EGFR. NONO was upregulated in 80 paired TNBC tissues and indicated a poor prognosis. Furthermore, NONO knockout significantly inhibited TNBC proliferation in vitro and in vivo. Mechanistically, NONO increased the stability of nuclear EGFR and recruited CREB binding protein (CBP) and its accompanying E1A binding protein p300, thereby enhancing the transcriptional activity of EGFR. In turn, EGFR positively regulated the affinity of NONO to mRNAs of nuclear EGFR downstream genes. Furthermore, the results indicated that the nuclear EGFR/NONO complex played a critical role in tumorigenesis and chemotherapy resistance. Taken together, our findings indicate that NONO enhances nuclear EGFR-mediated tumorigenesis and may be a potential therapeutic target for TNBC patients with nuclear EGFR expression.

## Introduction

Approximately 15% of all diagnosed breast cancers are triple-negative breast cancer (TNBC). Up to 95% of TNBCs are classified as invasive mammary carcinoma with a high rate of recurrence and poor overall survival. It is difficult to target therapeutically owing to the absence of biomarkers (ER/PR/HER2) [1–3].

Gene-expression profiling studies indicate about 25–50% of TNBCs overexpress epidermal growth factor receptor (EGFR). However, clinical trials failed to demonstrate significant activity of EGFR-targeted monoclonal antibodies and/or tyrosine-kinase inhibitors [4]. Many mechanisms of resistance to EGFR inhibitors have been discovered, one of which can be attributed to its location from the cell membrane to the nucleus [5, 6]. The constitutive presence of EGFR in the tumor cell nucleus has been reported to be a poor prognostic indicator of invasive breast cancer [7]. Inside the nucleus, EGFR functions as a transcriptional mediator to activate the expression of cyclin D1 [8], B-Myb [9], COX-2 [10], Aurora A [11], c-Myc [12], BCRP [13], and iNOS [14], also as a tyrosine kinase to phosphorylate and stabilize PCNA [15] and participated in DNA repair [16]. Moreover, these well-known targets are closely related to malignant phenotypes of TNBC, including tumorigenesis [17], cancer stemness [18, 19], metabolic reprogramming [20], and drug resistance [21], etc. Therefore, novel combination treatments that can overcome nuclear EGFR-mediated therapeutic resistance seem to be a promising anticancer strategy for TNBC. However, anticancer treatments on the extent of EGFR nuclear translocation are still limited and confusing. For example, Dittmann et al. reported cetuximab could inhibit radiation-induced EGFR nuclear transport [22]. In contrast, Liao and Carpenter showed that cetuximab promoted EGFR nuclear transport by activating receptor endocytosis and subsequent intracellular trafficking [23]. The study of nuclear EGFR is still in its infancy.

To clarify the molecular mechanism of nuclear EGFR in TNBC, gene-annotation enrichment analysis of the interactors of nuclear EGFR was performed, and the results showed that these interactors significantly enriched in RNA-binding. RBPs are particularly relevant to posttranscriptional modulation in virtue of structural and functional diversity. Mounting evidence has shown that RBPs are involved in various important biological processes [24]. Moreover, recent studies have identified RBPs play a critical role in TNBC [25, 26], but the underlying mechanisms and clinical significance of the vast majority of RBPs in TNBC remain unknown. Here, we observed non-POU domain-containing octamer-binding protein (NONO, a.k.a. p54^nrb^) significantly interacted with nuclear EGFR. NONO belongs to the Drosophila behavior/human splicing (DBHS) family, a typical RBP via its two highly conserved RNA-recognition motifs. The emerging paradigm describes NONO as a “multipurpose molecular scaffold”, as it engages in almost every step of gene regulation, including transcriptional activation and inhibition, RNA process, and DNA repair [27]. We previously reported that NONO supported breast cancer growth by regulating SREBP-1-mediated lipogenesis [28]. However, little is known about the functional role and molecular mechanism of NONO in nuclear EGFR-mediated TNBC progression.

Here, we found EGFR/NONO complex in the nucleus accelerated the TNBC tumorigenesis. Mechanistically, NONO promoted transcription of nuclear EGFR target genes by stabilizing nuclear EGFR and recruiting the transcriptional co-activator CBP/p300. Moreover, EGFR could enhance the affinity of NONO to its downstream transcripts. Our findings indicated that NONO played an important role in nuclear EGFR-mediated tumorigenesis and might be a potential therapeutic target for TNBC patients with EGFR nuclear localization.

## Materials and methods

### Cell lines and cell culture

The human breast cancer lines MCF-10A, MCF-7, T47D, MDA-MB-231, MDA-MB-468, and MDA-MB-453 were obtained from ATCC and DSMZ, cultured in Dulbecco’s modified Eagle’s medium supplemented with 10% fetal bovine serum, 100 µg/mL penicillin, and 100 µg/mL streptomycin (Gibco, Grand Island, NY, USA) at 37 °C. These cell lines were authenticated by short tandem repeat analysis and tested negative in mycoplasma tests.

### Antibodies and reagents

The antibodies used in this study were listed in Table S1. All untrimmed western blotting images were supplemented in original data. The following additional reagents were in the present study: MG132 (cat. no. HY-13259, MCE, USA); cycloheximide (cat. no. HY-12320, MCE, USA); cisplatin (cat. no. HY-17394, MCE, USA); doxorubicin (CSN16255, CSNpharm, China).

### Mining interactors of nuclear EGFR

The interactors of human EGFR were downloaded from BioGRID4.3 (https://thebiogrid.org/) [29], STRING (https://string-db.org/) [30] databases. Then, the Cellular Component of Gene Ontology (GO-CC) was used to analyze the subcellular location of those interactors by using DAVID v6.8 (https://david.ncifcrf.gov/) after merging duplicates. Thus, the interactors of EGFR in the nucleus were collected to perform Molecular Component of Gene Ontology (GO-MF) analysis via DAVID v6.8. The result was visualized by using R (v3.5.1) software based on GOplot2 package (https://cran.r-project.org/web/packages/GOplot/vignettes/GOplot_vignette.html).

### Data mining and analyzing

Several statistical mining tools were used to comprehensively characterize the clinicopathological characteristics of the interactors of nuclear EGFR in TNBC. UALCAN (http://ualcan.path.uab.edu/) [31] was used to assess the expression of those interactors in TNBC. Kaplan–Meier plotter (http://kmplot.com/analysis/) [32] was used to evaluate their prognostic merit in breast cancer. Breast Cancer Gene-Expression Miner v4.5 (http://bcgenex.centregauducheau.fr/bc-gem/gem-accueil.php?js=1) [33] was used to examine their expression and effects for survival in breast cancer.

### Subcellular fractionation and Co-immunoprecipitation (Co-IP)

Nuclear and cytosolic protein was extracted by using Minute^TM^ Nuclear and Cytoplasmic Extraction kit (Invent Biotechnologies) according to the manufacturer’s instructions. Alpha-tubulin and Lamin B1 were used as cytoplasmic and nuclear controls, respectively.

Beads were incubated with indicated antibody (or IgG as negative control) for 30 min at room temperature, then mixed with indicated cell lysate overnight at 4 °C, respectively. After washing three times with IP buffer, the samples were analyzed by immunoblotting.

### Proximity ligation assay (PLA)

PLA was performed by using the Duolink^®^ In situ Red Starter Kit Mouse/Rabbit (Sigma-Aldrich). Briefly, cells were grown on glass coverslips, fixed with 4% paraformaldehyde for 15 min, permeabilized with 0.1% Triton X-100 for 10 min, blocked with Block solution for 1 h. Next, primary and secondary antibodies with PLA probes (PLUS and MINUS) were added in sequence. After incubating with antibodies, the sample was incubated with ligation buffer with ligation enzyme. Next, polymerase was used to amplify the DNA circle. Finally, coverslips were mounted on the slide with Duolink^®^ In situ Mounting Medium with DAPI. Slides were analyzed by confocal micros + copy (Leica TCS SP8) using a 63× objective.

### Molecular docking

Molecular docking was performed as previously reported [34]. Briefly, human SFPQ (PDB ID: 4WIJ) was selected as templates for NONO. Human SFPQ (PDB ID: 4WIJ) and EGFR (PDB ID:1M14) were downloaded from PDB (http://www.rcsb.org/). All crystallographic water and other small molecules were removed. ZDOCK web server (http://zdock.umassmed.edu/) was used for molecular docking analyses. Binding affinities for those complexes were evaluated by ZDOCK 2.3.2 scoring function. High scores mean high affinity. The docking structures were visualized by PyMOL software v1.3 (DeLanoScientific, San Carlos, CA, US).

### GST pull-down assay

GST pull-down assays were performed as previously described [34]. Briefly, the GST-fused EGFR or NONO full-length and fragments were expressed in E. coli BL21 and purified using glutathione-agarose beads (GE healthcare, Uppsala, Sweden). A few proteins were used to verify its expression by Coomassie blue staining. MDA-MB-231 cell lysates were incubated with GST or GST-fusion protein beads overnight at 4 °C. After washing with IP buffer 10 times, the beads were resuspended in loading buffer and subjected to western blot analysis.

### Plasmid, siRNA, and transfection

The open reading frame of EGFR and NONO were constructed into pEGP 3 × NLS vector (Genechem, Shanghai, China) and pcDNA3.1 vector (Invitrogen, USA), respectively. All plasmids were confirmed by DNA sequencing. SiRNA and negative control were designed and synthesized by GenePharma (Shanghai, China). Cells were transfected with plasmid and siRNA by using Neofect™ DNA transfection reagent (Neofect biotech, Beijing, China) and lipofectamine 2000 Reagent (Invitrogen, CA, USA), respectively. Cells were collected to perform functional assays after transfection 48 h. The sequence of siRNA oligos in our study was listed in Table S2.

### RNA-seq

Total RNA of cells with NONO knockdown was extracted using TRIzol (Ambion,15596-026) was described in our published article [34]. RNA sequencing was carried out in collaboration with Shanghai Oebiotech. *P* value <0.05 and fold change ≥2.0 were set as the threshold for significantly differential expression genes (DEGs). GO and pathway enrichment analysis for DEGs were also analyzed were performed by using DVAID 6.8 and figured based on the R package.

### CRISPR/Cas9-mediated knockout of NONO

The CRISPR/Cas9 system was used to knockout NONO as previously described [34]. Briefly, the single guide RNA (sgRNA) targeted coding sequence (CDS) of NONO was obtained from the published literature [35] and cloned into plenti-CRISPR v2 (a generous gift from Dr. Feng Zhang). Lentivirus was packaged in 293T cells and infected breast cancer cell lines following the standard instruction. Single colonies were isolated and validated by immunoblotting. Clone 2 was obtained from pLentiCRISPRv2-NONO-gRNA#4 and clone 3 was obtained from gRNA-NONO#40.

### Cell viability assays

The cell viability was detected by the colony formation, Cell-Counting Kit-8 (CCK-8) (Yeasen, Shanghai, China), and Ethynyldeoxyuridine analysis (Beytime, Nantong, China) as previously reported [36].

For colony formation, cells with NONO knockout or not were placed on a 6-well plate and cultured for 1 week. Then, the cloned cells were washed with PBS three times, fixed with 4% paraformaldehyde for 20 min, and stained with 0.1% crystal violet for 30 min. Finally, cells were washed with PBS, dried and photographed. And the number of clones was recorded.

For the CCK-8 assay, cells with NONO knockout or not were seeded on the 96-well plate. After adding 10 μL of CCK-8 solution, cells were incubated at 37 °C for 1 h and absorbance at the wavelength of 450 nm was detected for the growth curve.

For the EdU assay, cells were incubated with BeyoClickTM EdU-488 (Beytime) at 37 °C for 2 h. Then, cells were fixed by 4% paraformaldehyde, permeated by 0.25% Triton X-100, incubated with prepared click reaction buffer at room temperature for 30 min, stained with DAPI. Cells were visualized under a fluorescence microscope (Olympus, Tokyo, Japan). The ratio of Edu-positive cells (green) to the total number of DAPI-positive cells (blue) was calculated to show the ability of cell proliferation.

### Animal experiments

Four-week-old BALB/c-nu/nu female mice were purchased from Silaike Experimental Animal Co., Ltd. (Shanghai, China). All experimental procedures using animals were following the guidelines provided by the Animal Ethics Committee of Renji Hospital of Shanghai Jiao Tong University School of Medicine. Mice were randomly grouped and injected subcutaneously with 1 × 10^7^ NONO knockout MDA-MB-231 cells within matrigel in the right flank, while control cells in the left flank. Similarly, nuclear EGFR-OE/Cas9 cells and nuclear EGFR-OE/NONO-KO cells were injected subcutaneously into the left and right flank of mice with 1 × 10^7^ cells per injection, respectively. The volume of tumors was measured with calipers and calculated by the formula: Volume = 1/2 × length × width^2^. All mice were euthanized 4 weeks after injection, and the subcutaneous tumors were harvested, weighed, and stained with H&E and indicated antibodies.

### Human samples and IHC analyses

A total of 80 paired paraffin-embedded TNBC tissues were acquired from the surgical specimen archives of Renji Hospital, School of Medicine, Shanghai Jiao tong University. All patients signed written informed consent. This study was approved by Renji Hospital, School of Medicine, Shanghai Jiao tong University, following the International Ethical Guidelines for Biomedical Research Involving Human Subjects (CIOMS). The samples were subjected to immunohistochemical analyses as previously reported [37]. The signal intensity of IHC was independently scored by two experimental researchers. The signal intensity was scored on a scale of 0–3 and the percentage of area with the score of 0 (<5%), 2 (6% to 25%), 2 (26% to 50%), 3 (51% to 75%), and 4 (>76%). Immunohistochemistry (IHC) score (0 to 12) was defined as the product of the intensity and percentage of cells. The sample was considered positive when the IHC score was greater than or equal to 4.

### CUT&RUN assay

Cleavage Under Targets&Release Using Nuclease (CUT&RUN) assay (Cell Signaling Technology, USA) was carried out following the standard instruction. Briefly, 1.0 × 10^5^ Cells were collected and resuspended with 1× wash buffer, immobilized on Concanavalin A, permeabilized by digitonin, incubated with NONO antibody and pAG-MNase fusion enzyme overnight at 4 °C. The pAG-MNase fusion enzyme was activated by adding pre-cooled Calcium Chloride. After incubating for 30 min, 1 × stop buffer was added and DNA fragments were released. Finally, DNA fragments were purified by DNA Purification Buffers and Spin Columns (Cell Signaling Technology, USA) and quantified by qPCR. PCR primers for the CUT&RUN assay were listed in Table S3.

### Luciferase reporter assay

Cyclin D1 promoter region containing nuclear EGFR putative binding area was constructed into pGL3-based vectors as previously reported [8]. Cyclin D1 promoter sequences plus Renilla luciferase reporter plasmid were transfected into MDA-MB-231 cells with NONO knockout or overexpression. After transfection 24 h, firefly and renilla luciferase activity were determined by a dual-luciferase reporter assay system (Promega, USA) according to the manufacturer’s instructions. The ratio of firefly luciferase to renilla activity was calculated for each of the triplicates.

### RNA immunoprecipitation (RIP) and RIP-seq

RIP assays with the anti-NONO antibody were performed according to the instruction of the Magna^TM^ RIP RNA-binding protein immunoprecipitation kit (Millipore, Massachusetts, USA) [34]. Briefly, 2 × 10^7^ cells were collected to lyse with RIP lysis buffer. After incubating with anti-NONO antibody or rabbit IgG, the protein A/G magnetic beads were mixed with the cell lysis overnight at 4 °C. NONO-RNA complexes were purified with RIP wash buffer. Finally, RNA was extracted and quantified by RT-qPCR. The primers for RIP were listed in Table S4.

RIP sequencing was carried out in collaboration with Shanghai Cloudseq as previously reported. The high-quality reads were aligned to the human reference genome (UCSC hg38) with hisat2 software. Then, guided by the Ensembl gtf gene-annotation file, cuffdiff software (part of cufflinks) was used to get the FPKM as the expression profiles of mRNAs, and fold change and *p* value were calculated based on FPKM, differentially expressed mRNAs were identified. GO and pathway enrichment analysis was also performed based on the differentially expressed mRNAs.

### mRNA stability assay and RT-qPCR

Cells at 80% confluence were treated with 2 μg/mL actinomycin-D (MCE) and collected at the indicated time points. Total RNA extracted by TRIzol Kit (Omega, Norcross, GA, USA) was quantified was performed using Nanodrop (Thermo Fisher Scientific, Waltham, MA, USA). Complementary DNA (cDNA) produced with the cDNA synthesis kit (Takara, Otsu, Japan) was quantified by qPCR with SYBR Green PCR Master Mix (Yishen, Shanghai, China) in a StepOnePlus RT-PCR system (Thermo Fisher Scientific). Primers for RT-qPCR were listed in Table S5.

### Statistical analyses

All data were represented as mean ± s.d. of three or more independent experiments. Student’s *t* test or one-way analysis of variance followed by Dunnett’s multiple comparisons test was performed to evaluate differences between two groups or more than two groups, respectively. Pearson’s correlation was performed to analyze the correlation among genes in mRNAs or protein levels. Survival rates were determined using the Kaplan–Meier method (log-rank test). *P* value of <0.05 was considered statistically significant. All statistical analyses and figures were generated using GraphPad Prism 7 software. One asterisk, two asterisks and three asterisks indicate *P* < 0.05; *P* < 0.01; *P* < 0.001, respectively.

## Results

### NONO interacts with nuclear EGFR

**Fig. 1:**
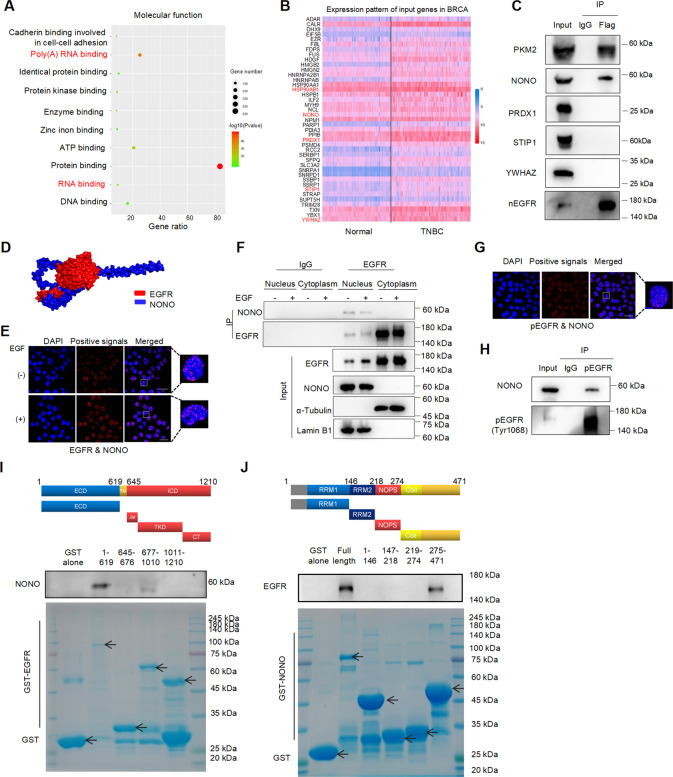
NONO interacts with nuclear EGFR. **A** The interactors of nuclear EGFR were downloaded from BioGRID^4.2^ (https://thebiogrid.org/) and STRING (https://string-db.org/) databases. Molecular function analysis showed these interactors were enriched in protein-binding, RNA binding, DNA binding, etc. **B** The heatmap showed the mRNA levels of those RBPs in normal breast and TNBC tissues via UALCAN portal-based TCGA dataset (http://ualcan.path.uab.edu/). **C** The association of nuclear EGFR with NONO, PRDX1, STIP1, YWHAZ in MDA-MB-468 cells was determined by western blotting. Nuclear lysates were extracted from MDA-MB-468 cells, then immunoprecipitated with an anti-EGFR antibody, finally loaded for western blotting with indicated antibodies. PKM2 as the positive control and IgG as negative control. nEGFR, nuclear EGFR. **D** The computational docking model for human EGFR (red) and NONO (blue) was predicted using ZDOCK software. **E** In situ proximity ligation assay (PLA) on MDA-MB-231 cells demonstrated the interaction between nuclear EGFR and NONO under EGF stimulus or not. Cells were serum-starved overnight before EGF treatment (100 ng/mL, 30 min). Positive PLA signals showed EGFR/NONO complex which was shown as red clusters, and cell nuclei were counterstained with blue (Scale bars = 10 μm). (−), without EGF treatment; (+), with EGF treatment. **F** Endogenous association of nuclear EGFR with NONO in MDA-MB-231 cells was detected by co-immunoprecipitation. Cells with 80-85% confluence were treated as (**E**). Then cells were collected and lysed. Equal amounts of cellular fractionated proteins were immunoprecipitated with an anti-EGFR antibody. Input samples from equal amounts of proteins blotted for indicated antibodies. (−), without EGF treatment; (+), with EGF treatment. **G** The interaction of phosphorated EGFR (Tyr1068) with NONO was detected by in situ proximity ligation assay in MDA-MB-231. Red clusters represented the phospho-EGFR/NONO complex. DAPI staining was used to determine the nuclei (Scale bars = 10 μm). pEGFR, phosphorated EGFR (Tyr1068). **H** Co-immunoprecipitation was used to examine the association of phosphorated EGFR and NONO. MDA-MB-468 cells were harvested and extracted nuclear fractions. Co-IP was carried out using the anti-phosphorated EGFR (Tyr1068) antibody. IgG as the negative control. **I**, **J** MDA-MB-231 cell lysates were incubated with GST-fusion proteins of the indicated fragments of EGFR (**I**) and NONO (**J**) in GST pull-down assays. All representative images of triplicate measurement results were similar and repeated 3 times.

To clarify the mechanism of nuclear EGFR, we investigated the nuclear interactome of EGFR obtained from BioGRID^4.3^ and STRING. GO molecular function annotation demonstrated these interactors were mainly enriched in poly(A) RNA-binding and protein-binding (Fig. 1A). Mounting evidence has shown that RNA-binding proteins (RBPs) engage in various biological processes of malignant cancers [38]. To assess the clinical significance of these RBPs in TNBC, we analyzed the TCGA dataset via UALCAN and found 41 RBPs significantly upregulated in TNBC (Fig. 1B). Further analysis showed the expression of HSP90AB1, NONO, PRDX1, STIP1, YWHAZ were higher in TNBC tissue compared with non-TNBC tissue (Fig. S1A–C) and positively correlated with poor overall survival rates (Fig. S1D–F). The correlation between EGFR and HSP90 has been widely studied [39, 40]. Therefore, we extracted nuclear cell lysis to examine the interaction of nuclear EGFR with NONO, PRDX1, STIP1, YWHAZ via co-immunoprecipitation. The result showed a strong protein-protein interaction between nuclear EGFR and NONO (Fig. 1C). The ZDOCK score for the docking conformation of the EGFR/NONO complex was 1509.5, suggesting a favorable and stable binding status (Fig. 1D). Furthermore, EGFR interacted with NONO in an EGF-independent manner (Fig. 1E, F) and phosphorylated EGFR was also associated with NONO (Fig. 1G, H). To confirm the structural determinants for the association between EGFR and NONO, GST pull-down assays were performed and showed that the extracellular domain (ECD) and tyrosine-kinase domain (TKD) of EGFR were necessary for their interaction (Fig. 1I). The same method was applied to map the NONO domain required for EGFR and found the coiled-coil domain was responsible for the interaction with EGFR (Fig. 1J). Interestingly, we also observed the EGFR/NONO complex in non-TNBC cell nuclei, but the complex was significantly less in non-TNBC cells (Fig. S2). These data indicate the constitutive presence of the EGFR-NONO complex in the tumor nucleus may contribute to malignant progression.

### NONO enhances proliferation of TNBC in vitro and in vivo

**Fig. 2:**
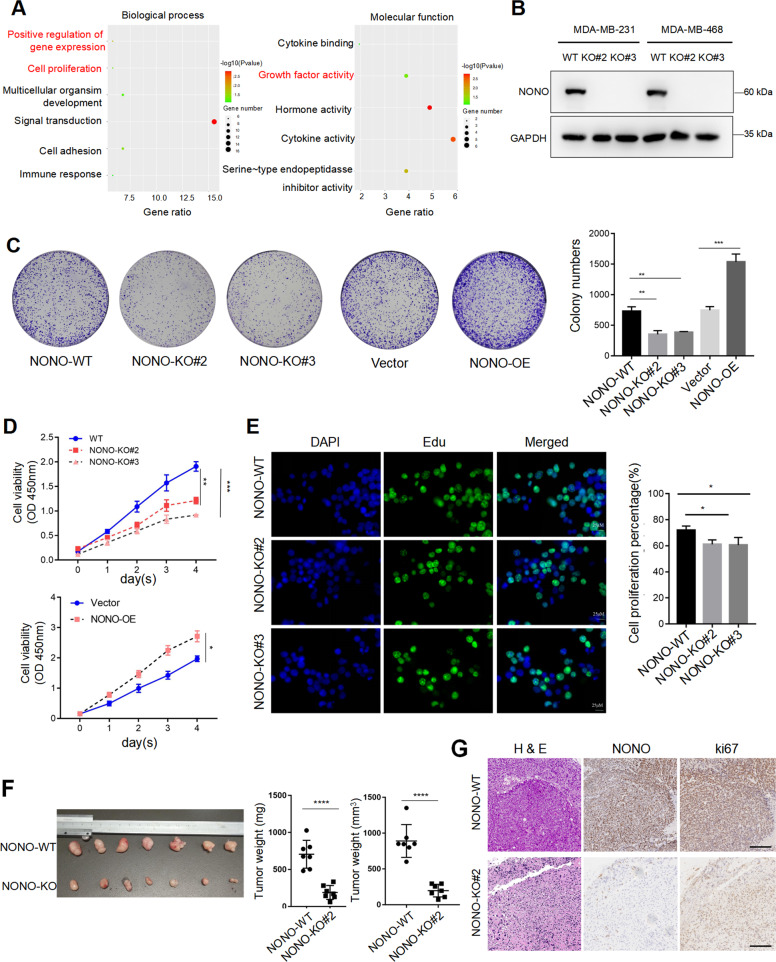
NONO functions as an oncogene enhancing proliferation of TNBC in vitro and in vivo. **A** GO analysis showed the differently regulated genes (*n* = 103) upon NONO knockdown were enriched in cell proliferation and growth factor activity. **B** The NONO knockout efficiency by CRISPR-Cas9 was confirmed by western blotting in MDA-MB-231 and MDA-MB-468 cells. **C**–**E** The effect of NONO on the cell viability of MDA-MB-231 was determined by colony formation (**C**), CCK-8 assay (**D**), and immunofluorescence analysis with Edu (**E**). Knockout of NONO expression significantly inhibited cell proliferation, while ectopic overexpression of NONO promoted cell proliferation. Scale bar = 100 μm (colony) or 20 μm (Edu). OE, overexpression. **F** The volume and weight of subcutaneous tumor was significantly diminished upon NONO knockout (*n* = 7 per group). **G** The xenografts were subjected to H&E and IHC staining with NONO, ki-67. Scale bar = 200 μm (×100). All values shown are mean ± SD of triplicate measurements and have been repeated three times with similar results (**C**–**E**). *P < 0.05; **P < 0.01; ***P < 0.01 versus corresponding control.

To elucidate the function of NONO, RNA-sequencing was performed and showed that NONO was closely associated with cell proliferation and growth factor activity (Fig. 2A). Next, CRISPR/Cas9 genome editing was used to generate Cas9 CRISPR KO NONO (NONO-KO) and Cas9 (NONO-WT) cells (Fig. 2B). The clone numbers of TNBC cells were strongly reduced in the absence of NONO, and vice versa (Fig. 2C). The results from CCK-8 and Edu assays further confirmed the result of clone formation (Fig. 2D, E). Finally, the mouse subcutaneous xenograft model was established to assess the effect of NONO on tumor growth in vivo. The weight and volume of subcutaneous tumors were significantly decreased upon NONO knockout (Fig. 2F). Consistently, IHC staining of tumor tissue indicated that NONO-silenced tumors had lower expression of ki-67 (Fig. 2G). Collectively, these results show that NONO promoted tumor growth in vitro and in vivo.

### NONO is upregulated in TNBC and associates with breast cancer malignancy

**Fig. 3:**
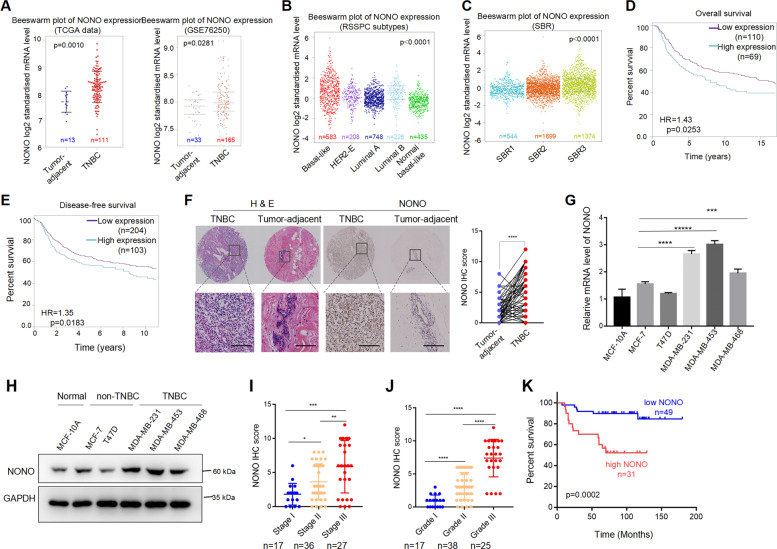
NONO is upregulated in TNBC and associates with breast cancer malignancy. **A** Beeswarm plots showed the NONO mRNA expression (Log_2_) levels in TNBC and tumor-adjacent tissues from TCGA-TNBC cohort (left) and GSE76250 (right). (TCGA: Tumor adjacent (*n* = 13) < TNBC (*n* = 111), *p* = 0.0010; GSE76250: Tumor adjacent (*n* = 33) < TNBC (*n* = 165), *p* = 0.0281). **B** Beeswarm plots showed the NONO mRNA expression (Log_2_) levels in different subtypes of breast cancer. (Basal-like, *n* = 583; HER2-E, *n* = 208; Luminal A, *n* = 748; Luminal B, *n* = 226; Normal breast-like, *n* = 435; Basal-like > Normal breast-like, *p* < 0.0001; Basal-like > luminal A, *p* < 0.0001; Basal-like > luminal B, *p* < 0.05; Basal-like > HER2-E, *p* < 0.001). **C** Beeswarm plots showed the NONO mRNA expression (Log2) levels in different tumor grade status. (SBR1, *n* = 544; SBR2, *n* = 1699; SBR3, *n* = 1374; SBR2 > SBR1, *p* < 0.0001; SBR3 > SBR1, *p* < 0.0001; SBR3 > SBR2, *p* < 0.0001). **D** Kaplan–Meier analysis of overall survival (OS) (log-rank, two sides) of TNBC patients with low (*n* = 110) or high (*n* = 69) mRNA level of NONO from bc-GenExMiner v4.5 (http://bcgenex.centregauducheau.fr/bc-gem/gem-accueil.php?js=1) based on DNA microarray data. **E** Kaplan–Meier analysis of disease-free survival (DFS) of TNBC patients with low (*n* = 204) or high (*n* = 103) mRNA level of NONO from bc-GenExMiner v4.5 based on DNA microarray data. **F** The quantitative expression of NONO protein in 80 paired TNBC tissues was analyzed by IHC. Representative H&E (left) and IHC staining (right) images showed the protein level of NONO in TNBC and paired adjacent normal breast tissue. Scale bar = 100 μm (×200). **G**, **H** The mRNA (**G**) and protein (**H**) level of NONO in different breast cancer cell lines. **I**, **J** The protein level of NONO in different tumor stages (**I**) and grades (**J**) of TNBC. **K** Kaplan–Meier analysis of overall survival of TNBC patients with low (*n* = 49) or high (*n* = 31) expression of NONO protein. The displayed values are the mean ± SD. The triplicate measurement results were repeated 3 times and the results were similar (**G**, **H**). *P < 0.05; **P < 0.01; ***P < 0.01 versus the corresponding control.

To assess the clinical significance of NONO in TNBC, we queried the TCGA, GEO databases and found NONO upregulated in TNBC compared with tumor-adjacent tissues (Fig. 3A). Moreover, the mRNA level of NONO was higher in the more aggressive basal-like TNBC subtype (Fig. 3B). Patients with higher mRNA expression of NONO had higher pathological grades (Fig. 3C) and shorter overall survival (OS) (Fig. 3D) and disease-free survival of TNBC (Fig. 3E). Immunohistochemistry (IHC) analysis of 80 paired TNBC and adjacent normal tissue sections further confirmed the clinical significance of NONO. NONO protein level in TNBC tissue was strikingly increased compared with the paired non-tumor tissues (Fig. 3F). Moreover, the expression of NONO was higher in TNBC cell lines than non-TNBC cell lines (Fig. 3G, H). Upregulated NONO positively correlated with advanced tumor stages and higher pathological grades (Fig. 3I, J and Table 1). Survival analysis showed that patients with higher NONO protein levels had a worse prognosis (Fig. 3K). In summary, our results show that NONO highly expresses in TNBC and closely associates with the malignancy of breast cancer.

**Table 1:** Analysis of correlation between NONO expression and clinic parameters of triple-negative breast cancer.

Characteristics	All cases	Low	High	*P* value
Participants	80	44	36	
Age (years)				0.8746
≤50	43	24	19	
>50	37	20	17	
Tumor size				
≤20 mm	31	26	5	<0.0001
>20 mm	49	18	31	
Lymph node metastasis				
Negative	37	27	10	0.0027
Positive	43	17	26	
Tumor stage				0.0002
I	17	15	2	
II	36	22	14	
III	27	7	20	
Tumor grade				<0.0001
1	17	17	0	
2	38	23	15	
3	25	4	21	

### NONO is required for the expression of nuclear EGFR targets

**Fig. 4:**
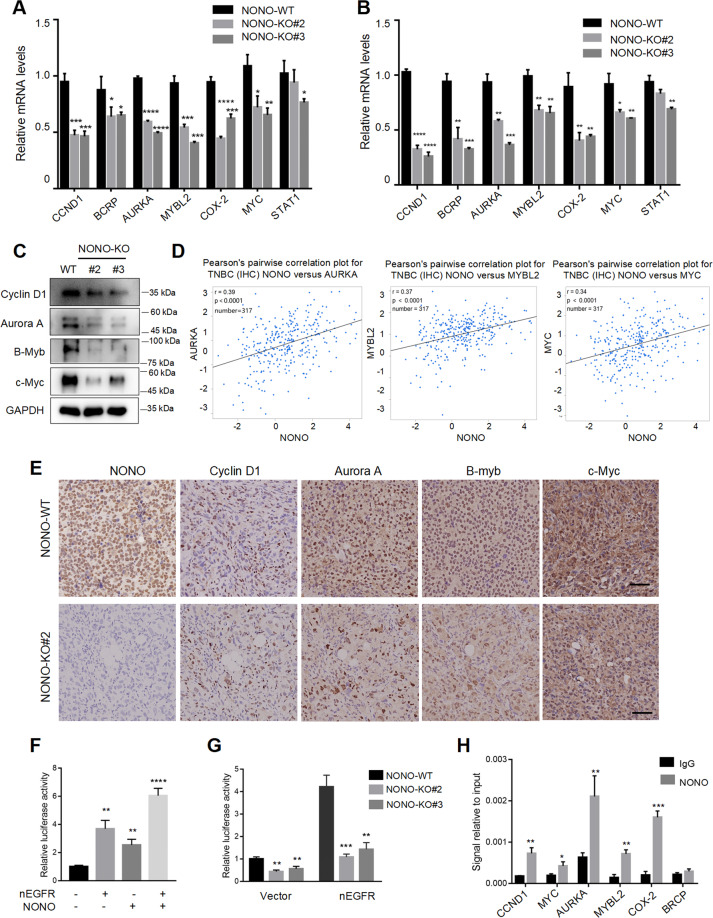
NONO is required for the expression of nuclear EGFR targets. **A**, **B** The mRNA of nuclear EGFR targets (CCND1, BCRP, AURKA, MYBL2, COX-2, MYC, STAT1) were strongly declined upon NONO knockout in MDA-MB-468 (**A**) and MDA-MB-231 (**B**). **C** The protein of nuclear EGFR targets (Cyclin D1, Aurora A, B-Myb and c-Myc) were significantly decreased in MDA-MB-231 cells upon NONO knockout. **D** The expressional correlation between NONO and nuclear EGFR target genes (AURKA, MYBL2 and MYC) in the TNBC (*n* = 317) were obtained from the bc-GenExMiner v4.5 database. The corresponding correlation plots were shown with Pearson’s coefficients. **E** The correlation between NONO and cylinD1, Aurora A, B-Myc, and c-Myc were shown in the representative IHC images of subcutaneous tumor tissue. Scale bar = 100 µm (×200). **F** Reporter assays were performed in MDA-MB-231 cells after being co-transfected with NONO and 3 × NLS EGFR plasmids for 48 h. Cells presented as a relative value with normalization against Renilla-Luc activity. nEGFR, nuclear EGFR. **G** Reporter assays performed in NONO knockout and control cells transfected with 3 × NLS EGFR or empty vector. nEGFR nuclear EGFR. **H** Cut&Run assay with NONO antibody was performed to verify the binding between NONO and promoters of nuclear EGFR targets in MDA-MB-468 cells. IgG as the negative control. Data are showed as the mean ± SD. The triplicate measurement results were repeated 3 times and the results were similar. *P < 0.05; **P < 0.01; ***P < 0.01; ****P < 0.01 as indicated.

As we know, nuclear EGFR behaves as a transcriptional regulator to promote the expression of target genes. And we found the transcriptional targets of nuclear EGFR were strongly decreased upon NONO knockout (Fig. 4A–C). What’s more, nuclear EGFR target genes AURKA (*r* = 0.39, *p* < 0.0001), MYBL2 (*r* = 0.37, *p* < 0.0001), and MYC (*r* = 0.34, *p* < 0.0001) showed a positive correlation with the expression of NONO in the TCGA-TNBC provisional cohort (Fig. 4D). Immunohistochemistry analysis of mouse subcutaneous tumor showed decreased expression of nuclear EGFR targets (cyclin D1, Aurora A, B-myb, c-Myc) in the absence of NONO (Fig. 4E), indicating that NONO was closely related to the nuclear EGFR transcription network. Furthermore, ectopic expression of EGFR or NONO alone increased the promoter activity of cyclin D1, while the co-expression of EGFR and NONO synergistically enhanced promoter activity (Fig. 4F). And NONO knockout resulted in a significant reduction in cyclin D1 transcriptional activity (Fig. 4G). We further found NONO could bind to the promoter region of the nuclear EGFR target genes by performing Cut&Run assay (Fig. 4H). Taken together, these data indicate that NONO enhances the transcriptional activity of nuclear EGFR and serves as a key regulator of the EGFR transcription network.

### NONO recruits CBP/p300 to the promoter of nuclear EGFR targets and stabilizes nuclear EGFR

**Fig. 5:**
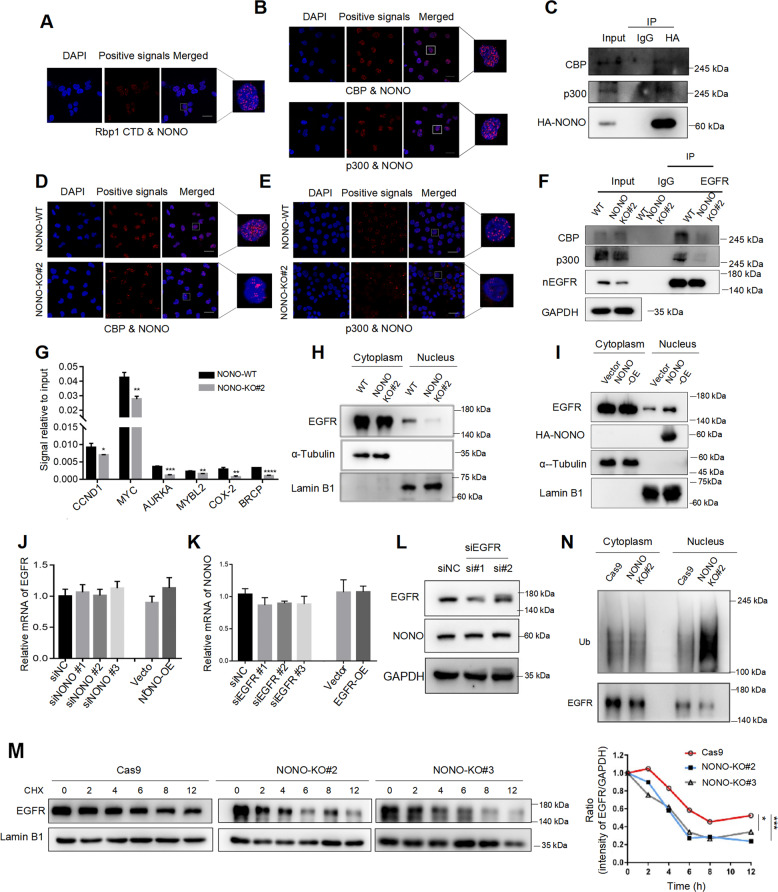
NONO recruits CBP/p300 to the promoter of nuclear EGFR targets and stabilizes nuclear EGFR. **A** In situ proximity ligation assay (PLA) on MDA-MB-231 cells demonstrated the interaction between NONO and the carboxy-terminal domain (CTD) of Rbp (Rbp-CTD). Positive PLA signals showed Rbp-CTD/NONO complex which was shown as red clusters, and cell nuclei were counterstained with blue (Scale bars = 10 μm). **B** In situ proximity ligation assay (PLA) on MDA-MB-468 cells indicated the interaction of NONO with CBP/p300. Positive PLA signals showed CBP/NONO (up) or p300/NONO (down) complex which was shown as red clusters, and cell nuclei were counterstained with blue (Scale bars = 10 μm). **C** Co-immunoprecipitation was used to detect the interaction of NONO with CBP/p300 in MDA-MB-231 after overexpressing HA-tag NONO. IgG as the negative control. **D**, **E** The association of NONO with CBP (**D**) or p300 (**E**) in MDA-MB-231 with NONO knockout or control cells was detected in situ proximity ligation assay (PLA). Positive PLA signals showed CBP/EGFR or p300/EGFR complex which was shown as red clusters, and cell nuclei were counterstained with blue (Scale bars = 10 μm). **F** Co-immunoprecipitation was used to detect the interaction of nuclear EGFR with CBP/p300 in MDA-MB-231 upon NONO knockout. IgG as the negative control. nEGFR nuclear EGFR. **G** Cut&Run assay with H3K18Ac antibody was performed to detect the acetylation level of histone 3 in the promoters of nuclear EGFR targets in MDA-MB-231 cells with NONO knockout or not. **H**, **I** Nuclear and cytosolic lysates were extracted from MDA-MB-231 cells with NONO knockout (**H**) or overexpression (**I**), followed by western blot analysis with indicated antibodies. **J** MDA-MB-231 cells were collected to detected mRNA of EGFR after being transfected with siNONO or NONO plasmid for 48 h. OE, overexpression. **K** After being transfected with siEGFR or EGFR plasmid for 48 h, MDA-MB-231 cells were collected to detected mRNA of NONO by RT-qPCR. **L** The protein level of NONO were detected in MDA-MB-231 with EGFR knockdown. **M** Nuclear lysates were extracted from MDA-MB-231 cells with NONO knockout or negative control following the treatment of cycloheximide (100 ng/ml) for the indicated time. The cell lysates were analyzed by immunoblotting. The relative level of EGFR was calculated by dividing the density of the EGFR signal by the corresponding Lamin B1 signal. Protein band intensity was analyzed by ImageJ. **N** Nuclear and cytosolic lysates were extracted from MDA-MB-231 cells with NONO knockout or negative control following MG132 treatment (100 μM, 4 h). Immunoprecipitation was performed with anti-EGFR beads, then ubiquitinated EGFR proteins were detected by immunoblotting. The representive images and triplicate measurement results were repeated 3 times and the results were similar. *P < 0.05; **P < 0.01; ***P < 0.01 versus the corresponding control.

In many cases, transcription activation by NONO involves the recruitment of RNA Polymerase II (RNAP II) [41]. We also found a strong interaction between NONO and the carboxy-terminal domain (CTD) of Rbp, the largest subunit of RNAP II (Fig. 5A). Besides the basal (general) transcription machinery, additional proteins are required to facilitate eukaryotic gene activation. We discovered that NONO interacted with CBP/p300 (Fig. 5B, C), which are typical chromatin-remodeling co-activator through their intrinsic histones acetyltransferases activity [42]. What’s more, knockout NONO significantly reduced the interaction between EGFR and CBP/p300 (Fig. 5D–F) and thus decreased the H3K18Ac level in the promoter of nuclear EGFR targets (Fig. 5G).

In addition, we found that nuclear EGFR protein expression was decreased upon knockout NONO (Fig. 5H) and enhanced with NONO overexpression (Fig. 5I), while the EGFR mRNA level was not significantly changed (Fig. 5J). And EGFR had no significant effect on the expression of NONO (Fig. 5K, L). Then we asked whether the protein-protein interaction increased the stability of nuclear EGFR and the cell was treated with cycloheximide to examine the degradation rate of nuclear EGFR. As shown in Fig. 5M, the nuclear EGFR degradation significantly accelerated in the absence of NONO. Consistently, NONO knockout caused a significant increase of ubiquitination of endogenous nuclear EGFR (Fig. 5N). These results demonstrated NONO promoted the expression of nuclear EGFR targets by recruiting CBP/p300 and stabilizing nuclear EGFR.

### Nuclear EGFR regulates the affinity of NONO to mRNAs

**Fig. 6:**
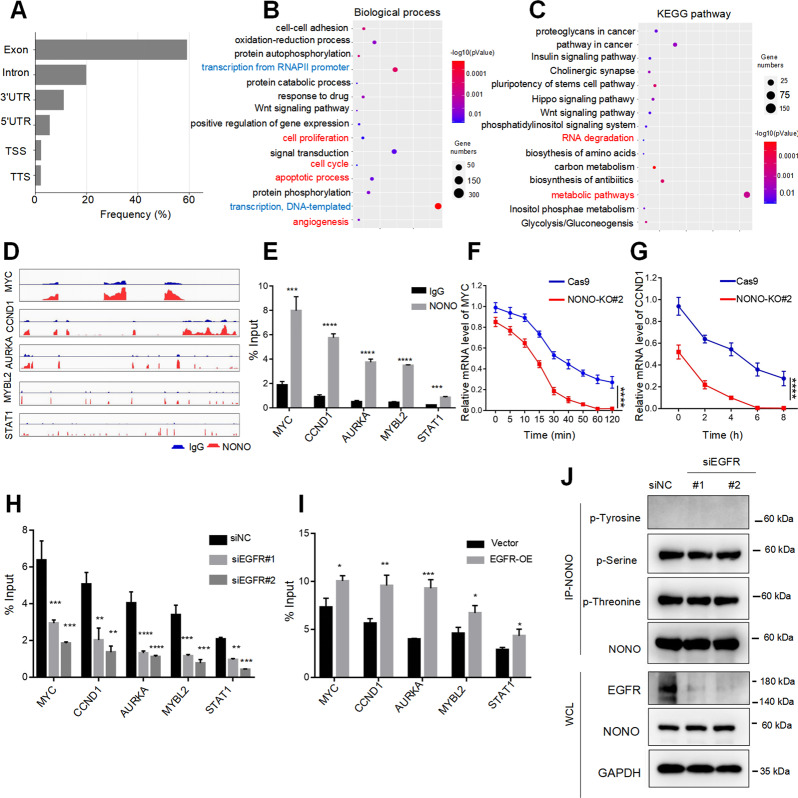
Nuclear EGFR regulates the affinity of NONO to mRNAs. **A** Distribution of NONO RIP-sequencing peak annotation for different regions. **B**, **C** Gene enrichment of biological process (**B**) and enriched KEGG pathways (**C**) analysis for the NONO-bound mRNA. **D** IGV software view of the RIP-seq reads mapping to MYC, CCND1, AURKA, MYBL2, STAT1 gene. **E** Lysate from MDA-MB-468 was subjected to RIP assay, then RIP assay products were extracted by Trizol and quantified by RT-qPCR with indicated pairs of primers. **F**, **G** MD-MB-231 cells were treated with actinomycin D (2 μM) and harvested at the indicated time points, then RNA was extracted from these cells and quantified by RT-qPCR with the indicated pairs of primers. **H**, **I** The effect of EGFR on the RNA-binding affinity of NONO was identified by knockdown (**H**) or overexpressing EGFR (**I**) in MDA-MB-231 cells. **J** The effect of EGFR knockdown on the phosphorylation of NONO in MDA-MB-231 cells. The displayed values are the mean ± standard deviation. The triplicate measurement results were repeated 3 times and the results were similar. *P < 0.05; **P < 0.01; ***P < 0.01 versus the corresponding control.

Next, we performed the RIP-seq to identify the NONO-associated transcriptome. Most identified RNAs bound by NONO were mature RNA (59.91%) and pre-mRNA (34.02%), with some noncoding RNAs (6.07%). And about 59.19% of NONO binding sites were within exons (Fig. 6A). GO enrichment analysis of molecular function showed these mRNAs bound by NONO enriched in nuclear EGFR-mediated phenotypes, including cell proliferation, cell cycle, apoptotic process and angiogenesis (Fig. 6B). KEGG-functional annotation revealed multiple pathways in cancer and metabolic pathways as major functional clusters (Fig. 6C). What’s more, the RIP-seq reads specially mapped to nuclear EGFR targets, including MYC, CCND1, AURKA, MYBL2, and STAT1 (Fig. 6D). RIP quantitative RT-PCR was confirmed the binding of NONO to these mRNAs (Fig. 6E). The reduced stability of MYC and CCND1 in NONO knockout cells was verified by RT-qPCR, using actinomycin D to inhibit transcription (Fig. 6F, G). Next, we explored whether its affinity to the nuclear EGFR targets was affected by EGFR. The results revealed that silencing of EGFR suppressed the interaction between NONO and transcripts of nuclear EGFR, and vice versa (Fig. 6H, I). Previous studies have reported the function of NONO could be likely post-translationally regulated. And nuclear EGFR always interacts and phosphorylates several proteins to contributes to cancer progression. Therefore, we examined phosphorylation of NONO upon EGFR knockdown but found no significant change (Fig. 6J). Taken together, the interaction between NONO and transcripts of nuclear EGFR targets could be affected by nuclear EGFR.

### Nuclear EGFR/NONO complex is important for the malignant progression of TNBC

**Fig. 7:**
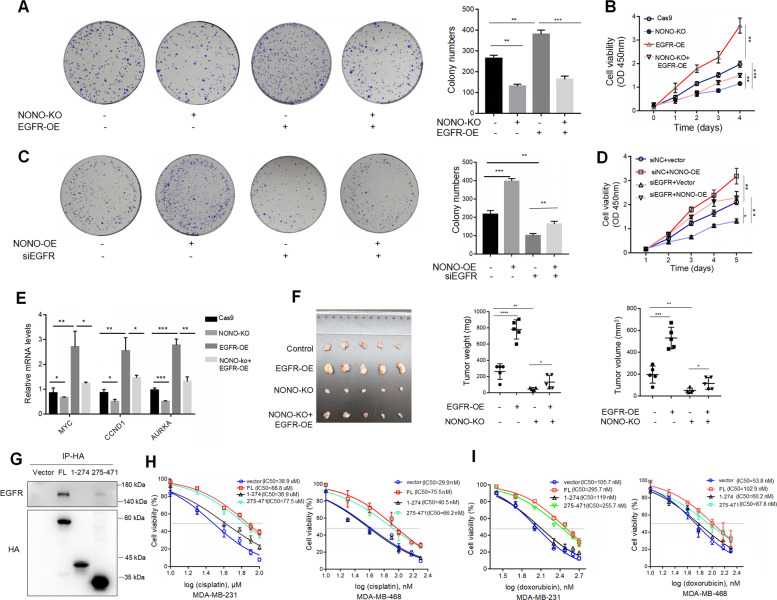
Nuclear EGFR/NONO complex is important for the malignant progression of TNBC. **A** Colony formation was used to analyze the effect of overexpressing EGFR on the cell viability of MDA-MB-468 with NONO knockout or not. **B** CCK-8 was used to analyze the effect of overexpressing EGFR on the cell proliferation of MDA-MB-231 with NONO knockout or not. **C** Colony formation was used to analyze the effect of overexpressing NONO on the cell viability of MDA-MB-468 with EGFR knockdown or not. **D** CCK-8 was used to analyze the effect of overexpressing NONO on the cell proliferation of MDA-MB-231 with EGFR knockdown or not. **E** RT-qPCR was used to analyze the effect of overexpressing EGFR on the mRNA levels of indicated genes in the absence of NONO in MDA-MB-231. **F** Image of xenograft tumors resected from tumor-bearing mice that injected subcutaneously with indicated cells (*n* = 5 per group). **G** The Functional domain of NONO responsible for the interaction with EGFR was detected by Co-IP. MDA-MB-231 cells with NONO knockout were transfected with full-length, aa 1-274, aa 275-471 fragments or vectors with HA-tag for 48 h, respectively. Then nuclear extracts were incubated with anti-HA beads overnight. Then endogenous EGFR was co-immunoprecipitated and detected by western blotting. **H** MDA-MB-231 (left) and MDA-MB-468 (right) with NONO knockout overexpressed different fragments of NONO, then treated with indicated concentrations of cisplatin for 24 h. The cells were then analyzed for CCK-8 assay and IC_50_ calculation. **I** MDA-MB-231 (left) and MDA-MB-468 (right) with NONO knockout overexpressed different fragments of NONO, then treated with indicated concentrations of doxorubicin for 24 h. The cells were then analyzed for CCK-8 assay and IC_50_ calculation. The displayed results are the mean ± SD. The triplicate measurement results were repeated 3 times and the results were similar. *P < 0.05; **P < 0.01; ***P < 0.01; ****P < 0.01 versus corresponding control.

Finally, we explored whether oncogenic functions of nuclear EGFR depended on NONO in TNBC. The results revealed that knockout NONO suppressed the cell viability stimulated by ectopic expression of EGFR (Fig. 7A, B). On the contrary, NONO overexpression could rescue the cell proliferation upon knockdown of EGFR (Fig. 7C, D). Consistently, decreased cell migration induced by NONO knockout could be rescued by EGFR overexpression (Fig. S3A, B). In addition, NONO knockout markedly reversed the expression of nuclear EGFR targets which were induced by EGFR overexpression (Fig. 7E). Importantly, the result of in vivo xenograft further confirmed that knockout NONO impeded nuclear EGFR-enhanced tumorigenesis (Fig. 7F). Nuclear location of EGFR has been implicated in drug resistance [43, 44]. We further examined the importance of nuclear EGFR/NONO in the drug resistance of TNBC. Compared with the full length and EGFR-binding domain (aa 274-471) of NONO (Fig. 7G), EGFR binding-deficient mutant (aa1-274) failed to induce cisplatin (Fig. 7H) and doxorubicin (Fig. 7I) resistance in TNBC, indicating the nuclear EGFR/NONO interaction also played important role in drug resistance. These results showed the oncogenic role of the nuclear EGFR/NONO axis in the malignant progression of TNBC.

## Discussion

**Fig. 8:**
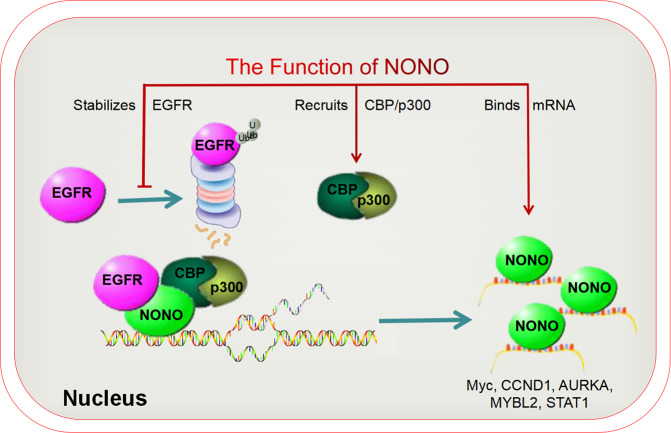
Schematic illustration of mechanisms of NONO in nuclear EGFR-mediated malignant progression of TNBC. On the one hand, NONO enhanced the transcription activity of nuclear EGFR by stabilizing its protein and recruiting the transcriptional co-activator CBP/p300. On the other hand, NONO bound subsequent mRNAs to enhance their stability, including Myc, CCND1, AURKA, and so on. What’s more, nuclear EGFR could enhance the affinity of NONO to mRNAs.

Nuclear translocation of full-length EGFR from plasma membrane has been recognized for ~30 years [45]. Mounting preclinical and clinical evidence supports the role of nuclear EGFR in the malignant progression of cancer and identified it as a predictor of shortened survival. In this study, we showed NONO engaged in nuclear EGFR-mediated tumorigenesis. On the one hand, NONO enhanced the transcription activity of nuclear EGFR by recruiting the transcriptional co-activator CBP/p300 and stabilizing nuclear EGFR protein, and bound subsequent mRNAs to enhance their stability. On the other hand, nuclear EGFR could enhance the affinity of NONO to mRNA (Fig. 8). Thus, even if EGFR was overexpressed with NONO knockout, tumor phenotype was partly restored.

To fundamentally understand the mechanism of EGFR, the EGFR interactome has been widely investigated in several types of cancers especially lung cancer [46, 47]. However, the majority of those researches focused on the EGFR-involved protein complex without taking subcellular distribution into account. Recently, Wang et al. investigated EGFR subcellular interactome in NSCLC cells and showed EGFR-interacting proteins in the nucleus involved in RNA processing [48]. Consistently, we queried several protein-protein interaction databases online and found that the nuclear interactors were mainly related to RNA binding. Both results consistently suggested modulating RNA metabolism was one of the main functions of nuclear EGFR.

Mainly in the past two decades, RBPs have already gained the sufficient attention of many researchers owing to their pivotal role in controlling the processing and transportation of RNA. Liu et al. reported DCAF3, a novel RBP, promoted TNBC metastasis by binding to and accelerating the degradation of DTX3 [25]. Chen et al. showed MCPIP1, a zinc finger RBP, suppressed the proliferation of TNBC by mediated NFIC alternative splicing [26]. To clarify the exact RBP in nuclear EGFR-mediated cancer progression, we evaluated the clinical value of those RBPs in TNBC and found NONO was significantly upregulated in TNBC and closely associated with breast cancer malignancy. de Silva et al. found that NONO was involved in IGFBP-3 mediated DNA damage repair in triple‑negative breast cancer [49]. Recently, Kim et al. reported that NONO interacted with and stabilized both mRNA and protein of STAT3, purposed NONO as a potential therapeutic target in TNBC [50]. Qin et al. showed that NONO formed a complex with moesin (MSN) to activate CREB signaling and might serve as a novel target for TNBC treatment [51]. These results confirm that NONO may be a promising therapeutic target for TNBC. Interestingly, we observed that NONO constitutively interacted with EGFR in the nucleus regardless of EGF stimulus. Moreover, NONO interacted with phosphorylated EGFR, the active form of EGFR. Nuclear presence of EGFR can be constitutive, in part, attributed to the EGF-EGFR autocrine loop thus initiating EGF-activated nuclear transport. Therefore, the constitutive presence of the EGFR/NONO complex in the tumor nucleus might suggest the malignant nature of cancer.

The multi-functional properties of NONO have been attributed to its associations with different protein partners. Here, we found NONO could recruit typical histones acetyltransferases CBP/p300 to promote gene transcription. Contrarily, several studies described that NONO could bind directly to target gene promoters, subsequently recruiting epigenetic silencers such as histone deacetylases (HDACs) for chromatin condensation and gene repression [52]. It seems that the gene-expression regulated by NONO becomes flexible through bidirectional regulation of histone acetylation level. Further work is needed to understand this duality of function, its modification status, cell-type-specific expression, cellular microenvironment and external stimulus should be taken into consideration.

As mentioned above, nuclear EGFR was closely related to RNA processing. Dittmann et al. observed nuclear EGFR interacted with and stabilized mRNAs by indirectly phosphorylating thus inhibiting the activity of PNPase, which was a negative regulator of mRNA stability [53]. In our study, we noticed that EGFR enhanced the affinity of NONO to the transcripts of nuclear EGFR target genes. Several studies reported post-translational modification of NONO affected its affinity to nucleic acid. Phosphorylation in N-terminal Thr15 residue of NONO induced by CDK1 could disrupt its interaction with RNA [54]. And methylation of conserved arginine residues in the coiled-coil domain negated the binding of NONO to mRNA [55]. Nevertheless, we found neither the serine nor threonine phosphorylation could be affected by EGFR. Additionally, no tyrosine phosphorylation of NONO was detected. Consistently, the crystal structure of NONO showed the Tyr residues of NONO were not accessible for protein kinases because of steric hindrance [56]. Other proteins might be involved in EGFR-mediated RNA-binding ability of NONO, which needed to be explored in future. Dittmann et al. reported mRNAs bound by nuclear EGFR in response to radiation enriched in Warburg effect and HIF-1a/VEGFA signaling [57]. Interestingly, gene-annotation of our RIP-seq revealed NONO involved in glycolysis and angiogenesis. We previously reported that NONO facilitated the hypoxia-enhanced glycolysis and angiogenesis by interacting with and pre-mRNA and subsequent mRNA of hypoxia-induced genes ^34^. These results suggested NONO was closely related to nuclear EGFR-mediated mRNA processing.

Even with mounting studies that have shown that nuclear EGFR functions as a true oncogene independently on its membrane-localized counterpart, no direct evidence demonstrate it can lead to tumorigenesis and/or cancer progression on its own. The answer to this question lies in the ability to especially interfere with nuclear EGFR. This feat is extremely hard to combat in the laboratory since nuclear-localized EGFR function is superimposed by the cytosolic signaling of membrane-associated EGFR [6, 58]. Our study also needs more direct results to interpret the mechanism of nuclear EGFR, the technical problem that needs to address in the future. Our findings suggested that nuclear EGFR/NONO positive feedback loop facilitates tumor malignant progression and NONO might be a promising therapeutic target for TNBC with high expression of nuclear EGFR.

## Supplementary information

supplementary data

Reproducibility checklist

## Data Availability

Raw sequencing and processed RNA Seq data from our study are not publicly available due to other unfinished researches but are available from the corresponding author on reasonable request.
