# Supplementary material for: RNA-binding protein p54nrb/NONO potentiates nuclear EGFR-mediated tumorigenesis of triple-negative breast cancer
Source: Cell Death Dis. 2022 Jan 10;13(1):42. doi: 10.1038/s41419-021-04488-9 (PMC8748691; doi:10.1038/s41419-021-04488-9)
Supplement: Supplementary file 1 — supplementary data [file 41419_2021_4488_MOESM1_ESM.doc]

**Supplementary figures**

**
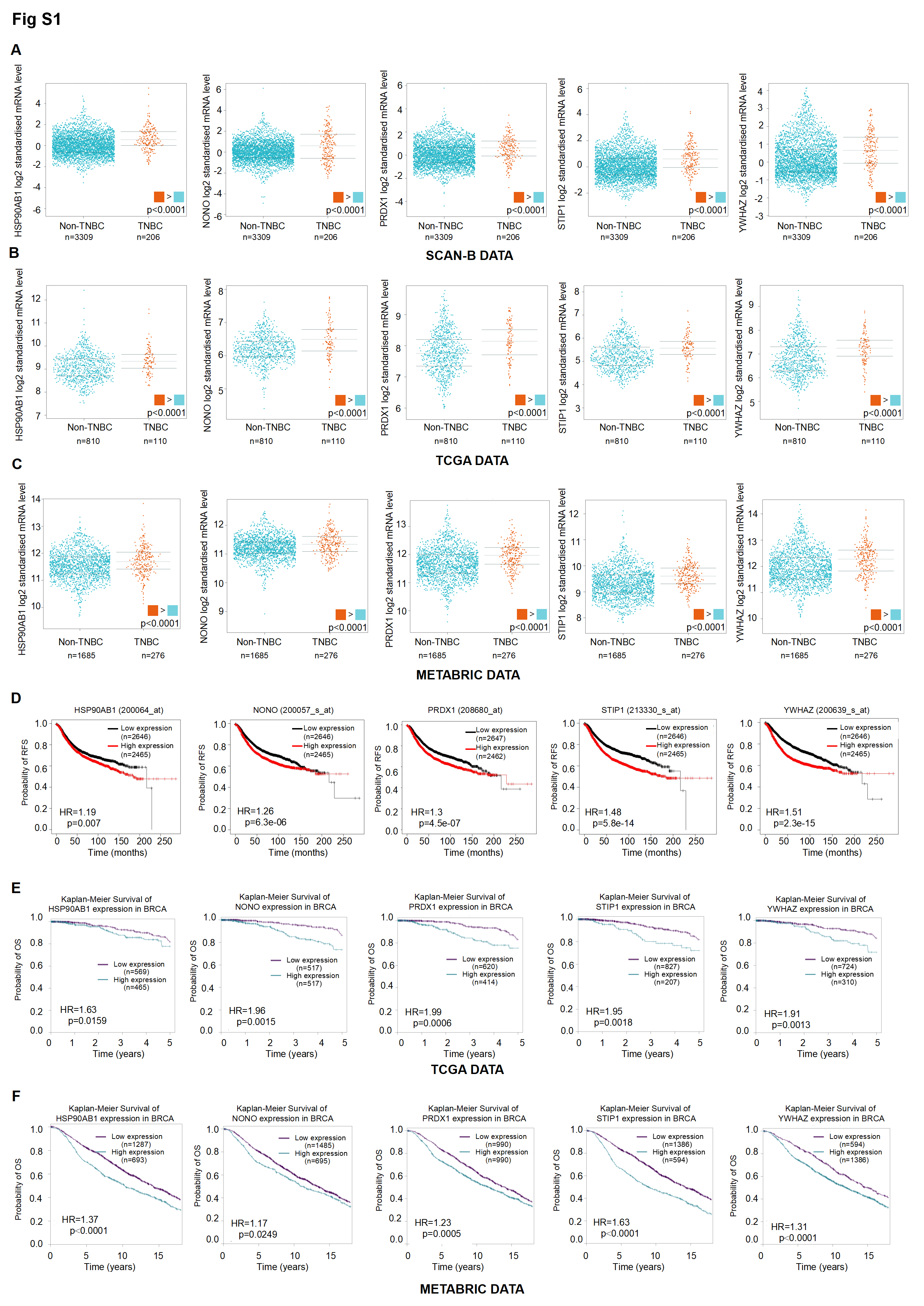
**

**Fig S1. Genes upregulated in TNBC and predicted poor prognosis.**

(A-C) Beeswarm plot showed the interactors of nuclear EGFR (HSP90AB1, NONO, PRDX1, STIP1, YWHAZ) were upregulated in TNBC tissue compared with non-TNBC tissue via bc-GenExMiner v4.5 based on SCAN-B (A), TCGA (B) and METABRIC (C) datasets.

(D) Kaplan-Meier analysis showed elevated the interactors of nuclear EGFR (HSP90AB1, NONO, PRDX1, STIP1, YWHAZ) were significantly associated with recurrence-free survival (RFS) of breast cancer via Kaplan-Meier plotter.

(E-F) Kaplan-Meier analysis showed upregulated the interactors of nuclear EGFR (HSP90AB1, NONO, PRDX1, STIP1, YWHAZ) were positively related with overall survival (OS) of breast cancer via bc-GenExMiner v4.5 based on TCGA (E) and METABRIC (F) datasets.

**
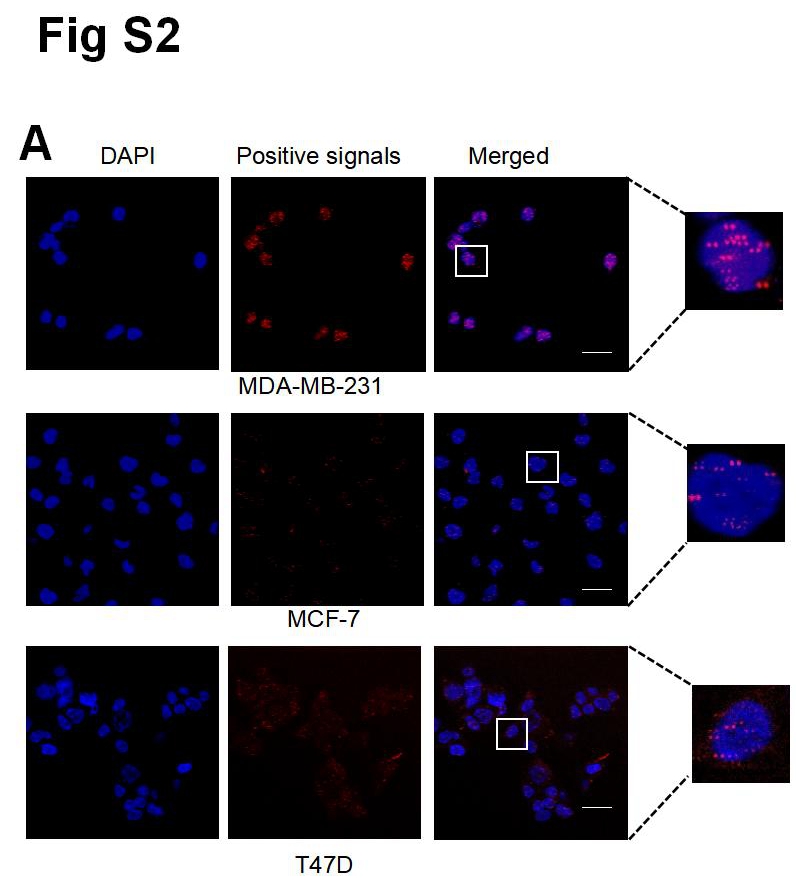
**

**Fig S2. The interaction of EGFR with NONO in breast cancer cells.**

1. In situ proximity ligation assay (PLA) on TNBC cells (MDA-MB-231) and non-TNBC cells (MCF-7 and T47D) demonstrated the interaction between the EGFR and NONO. Positive PLA signals showed EGFR/NONO complex which were shown as red clusters, and cell nuclei were counterstained with blue (Scale bars =10 μm). All representative images of triplicate measurement results were similar and repeated 3 times.

**
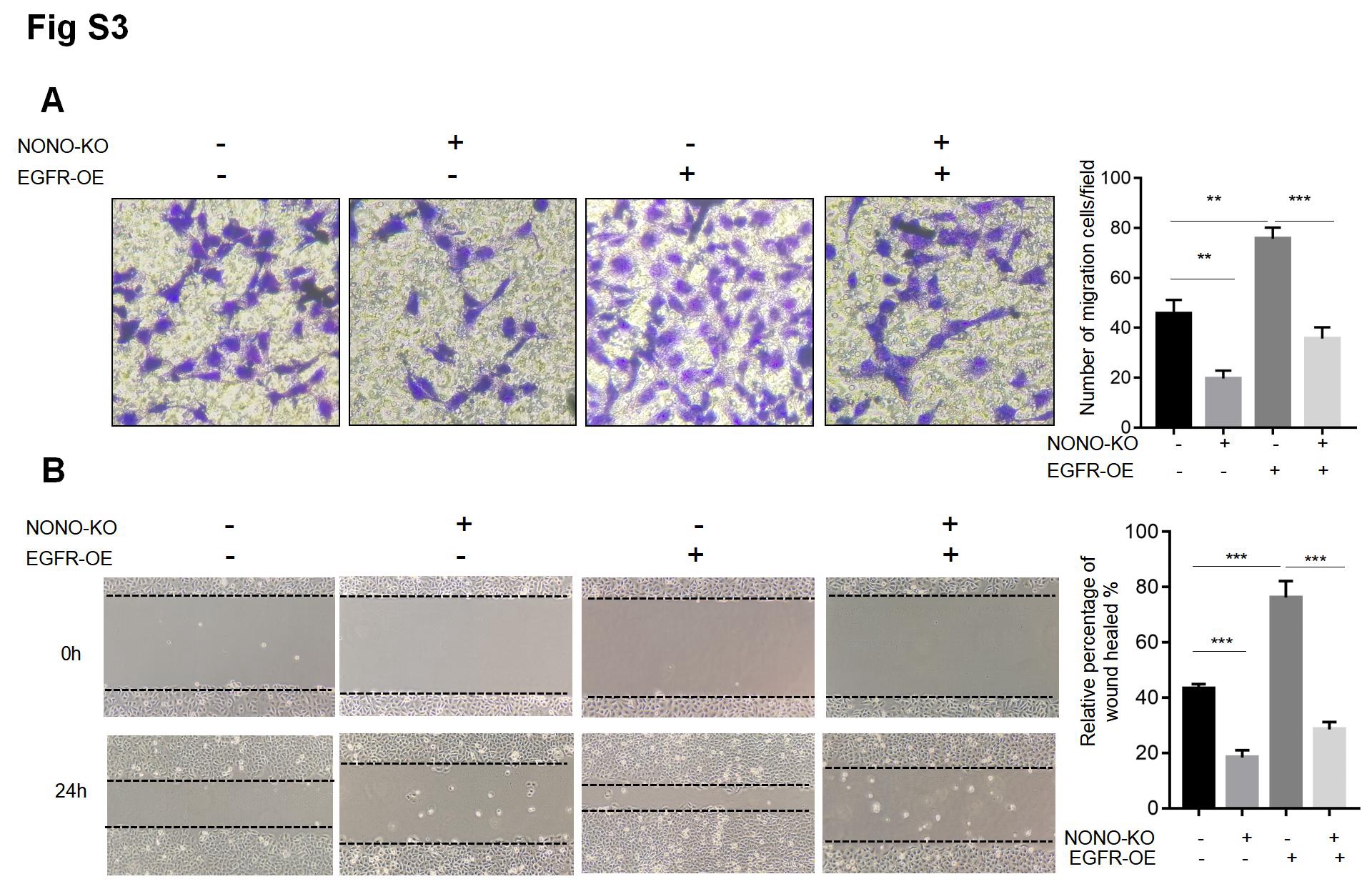
**

**Fig S3. The effect of nEGFR/NONO axis on the migratory ability of TNBC cells.**

1. B) Trans-well (A) and wound-healing (B) assays were used to analyze the effect of EGFR on the cell migration in the absence of NONO in MDA-MB-231. The displayed results are the mean±SD. The triplicate measurement results were repeated 3 times and the results were similar. *P < 0.05, **P < 0.01, ***P < 0.01, ****P < 0.01 versus corresponding control.

**Supplementary Tables**

**Table S1. Antibody list**

| Antibody | Source | Catalog Number | Application in our study |
| --- | --- | --- | --- |
| NONO | [Bethyl Laboratories](http://www.bethyl.com/) | A300-587A | WB, PLA, Cut&Run, RIP, RIP-Seq |
|  | [Santa Cruz Biotechnology](http://www.scbt.com/) | sc-376865 | PLA |
| EGFR | Cell Signaling Technology | 2085 | WB, PLA |
|  | [Santa Cruz Biotechnology](http://www.scbt.com/) | sc-120 | WB, PLA |
| pEGFR | Cell Signaling Technology | 2236 | WB, IP, PLA |
| p300 | Cell Signaling Technology | 86377s | WB, PLA |
| CBP | Cell Signaling Technology | 7389s | WB, PLA |
| H3K18Ac | MillporeSigma | 07354 | Cut&Run |
| HA-tag | Biolegend | 901503 | WB, IP |
| c-Myc | Proteintech | 10828-1-AP | WB, IHC |
| B-Myb | Proteintech | 18896-1-AP | WB, IHC |
| AuroraA | Abcam | ab13824 | WB, IHC |
| Cyclin D1 | Abcam | Ab16663 | WB, IHC |
| PKM2 | Cell Signaling Technology | 4053 | WB |
| STIP1 | Abcam | ab115730 | WB |
| PRDX1 | Proteintech | 15816-1-AP | WB |
| YWHAZ | Proteintech | 14881-1-AP | WB |
| Rpb1 CTD | Cell Signaling Technology | 2629 | PLA |
| Phospho-Tyrosine | Cell Signaling Technology | 8954 | WB |
| Phospho-Serine | Abcam | ab9332 | WB |
| Phospho-Threosine | Abcam | ab9337 | WB |
| Lamin B1 | Cell Signaling Technology | 13435 | WB |
|  | Proteintech | 12987-1-AP | WB |
| α-Tubulin | Proteintech | 11224-1-AP | WB |
| GAPDH | Proteintech | 60004-1-Ig | WB |
| Anti-Rabbit | Proteintech | SA00001-2 | WB |
|  | Abbkine | A25022 | WB (for COIP sample) |
| Anti-Mouse | Proteintech | SA00001-1 | WB |

**Table S2. siRNA sequence**

| Type | Sequence (5’-3’) |
| --- | --- |
| siNONO#1 | CAGGCGAAGUCUUCAUUCAUA |
|  | UAUGAAUGAAGACUUCGCCUG |
| siNONO#2 | GCCAGAAUUCUACCCUGGAAA |
|  | UUUCCAGGGUAGAAUUCUGGC |
| siNONO#3 | UCCAGAGAAGCUGGUUAUAAA |
|  | UUUAUAACCAGCUUCUCUGGA |
| siEGFR#1 | CCCGUCGCUAUCAAGGAAUUA |
|  | UAAUUCCUUGAUAGCGACGGG |
| siEGFR#2 | GCUGCUCUGAAAUCUCCUUUA |
|  | UAAAGGAGAUUUCAGAGCAGC |

**Table S3. Primers for CUT&Run**

| Gene | Primers (5’-3’) |
| --- | --- |
| MYC | F: CCTCCCGGGTTCCCAAAGCAG |
|  | R: CCAAGCCTCTGAGAAGCCCTGC |
| CCND1 | F: GGCAGAGGGGACTAATATTTCCAGCA |
|  | R: GAATGGAAAGCTGAGAAACAGTGATCTCC |
| AURKA | F: GGTTCAAGGAGGTCAGGACA |
|  | R: GGTGCCCTCAGTTCAAGGTA |
| MYBL2 | F: CTGGTCTTAGCTACCCGTGAGTTGA |
|  | R: CAGGAGTATCCCACATAGCGAACAC |
| STAT1 | F: GCTTCCGAGCTGTCAAGTAAAGTGAG |
|  | R: AAGCCGGCGGAAATACCCCA |
| COX-2 | F: AAGGGGAGAGGAGGGAAAAATTTGTG |
|  | R: GAGGCGCTGCTGAGGAGTTCCTG |
| BCRP | F: CCCGTCTCTATTTAAAATACACACAC |
|  | R: ATCTAAAGAGGACCGAGAAGC |

**Table S4. Primers for RIP**

| Gene | Primers (5’-3’) |
| --- | --- |
| MYC | F: AGCTTGTACCTGCAGGATCTGAGC |
|  | R: CAGAGTCGCTGCTGGTGGTG |
| CCND1 | F: GCTACAGATGATAGAGGAT |
|  | R: CTCTTCTACTTTAAAAATGGTTTG |
| AURKA | F: GCTACAGCTCCAGTTGGAGGT |
|  | R: GGCTGCTTGCTCTTTTGGGT |
| MYBL2 | F: GACTGCAGTTCCTGCGAGCGAGGAG |
|  | R: CAGCGCGTCCGCCGAGACATC |
| STAT1 | F: GCTCAGTCGGGGAATATTCAG |
|  | R: TCTTGAGTAACAGCTGTTCTTGTT |

**Table S5. Primers for RT-qPCR**

| Gene | Primers (5’-3’) |
| --- | --- |
| NONO | F: CGGGATCCTGCAGAGTAATAAAACTTTTAAC |
|  | R: GGAATTCAGTATCGGCGACGTTTG |
| EGFR | F: AGGCACGAGTAACAAGCTCAC |
|  | R: ATGAGGACATAACCAGCCACC |
| MYC | F: GTCAAGAGGCGAACACACAAC |
|  | R: TTGGACGGACAGGATGTATGC |
| CCND1 | F: GCTGCGAAGTGGAAACCATC |
|  | R: CCTCCTTCTGCACACATTTGAA |
| AURKA | F: GAGGTCCAAAACGTGTTCTCG |
|  | R: ACAGGATGAGGTACACTGGTTG |
| MYBL2 | F: CCGGAGCAGAGGGATAGCA |
|  | R: CAGTGCGGTTAGGGAAGTGG |
| STAT1 | F: CAGCTTGACTCAAAATTCCTGGA |
|  | R: TGAAGATTACGCTTGCTTTTCCT |
| COX2 | F: ATGCTGACTATGGCTACAAAAGC |
|  | R: TCGGGCAATCATCAGGCAC |
| BCRP | F: TGAGCCTACAACTGGCTTAGA |
|  | R:CCCTGCTTAGACATCCTTTTCAG |
